# Factors associated with acquisition of glycopeptide-resistant enterococci during a single-strain outbreak

**DOI:** 10.1017/S0950268818003655

**Published:** 2019-03-20

**Authors:** S. Deboscker, P. Schneider, F. Séverac, C. Ménard, J. Gaudart, T. Lavigne, N. Meyer

**Affiliations:** 1 Service d'hygiène hospitalière, Hôpitaux universitaires de Strasbourg, Strasbourg, France; 2 ICube, UMR7357, Université de Strasbourg, Strasbourg, France; 3 Pharmacie, Hôpitaux universitaires de Strasbourg, Strasbourg, France; 4 Groupe Méthode en Recherche Clinique (GMRC), Service de Santé Publique, Hôpitaux universitaires de Strasbourg, Strasbourg, France; 5 Laboratoire de bactériologie, Hôpitaux universitaires de Strasbourg, Strasbourg, France; 6 Hôpital La Timone, Service Biostatistique et Technologies de l'Information et de la Communication, APHM, Marseille, France; 7 IRD, INSERM, SESSTIM UMR912, Aix Marseille Univ, Marseille, France; 8 EA7290, Virulence bactérienne précoce, Fédération de médecine translationnelle de Strasbourg, Université de Strasbourg, Strasbourg, France

**Keywords:** Bayesian approach, case–control study, emerging micro-organism, glycopeptide-resistant enterococci (GRE), outbreak

## Abstract

The aim of our study was to describe and to investigate the factors associated with glycopeptide-resistant enterococci (GRE) acquisition during a single-strain outbreak which occurred in several wards of hospital from September 2013 to January 2014. We designed a case–control study. Analyses were performed using Bayesian methods. Univariate logistic regressions with informative priors from published studies were conducted. A multivariate model was build including variables with a probability of odd-ratio exceeding one (Pr) >85% or <15%. Thirteen cases and 52 controls were recruited. The description of this outbreak highlighted the importance to quickly detect patients at risk of GRE carriage in order to implement the isolation measures and to transfer to dedicated department if they are effectively carriers. Following multivariate analysis, antibiotics during hospitalisation (Pr = 0.968), number of hospitalisation days in the year (Pr = 0.964), antacids intake (Pr = 0.878) (with a risk increase), immunosuppression (Pr = 0.026) and isolation measures (Pr = 0.003) (both with protective effect) were associated with GRE acquisition. The use of Bayesian statistics was useful because of our study's small population size and prior information availability.

## Introduction

In French hospitals, the first outbreaks of glycopeptide-resistant enterococci (GRE) were reported in 2004–2005, then other reports followed [1–3]. Nevertheless, contrary to several European countries in which the spread of GRE is now endemic, their diffusion remains limited in France [4]. While several studies have shown an increase in GRE-related mortality and costs [5, 6], the risk is more ecological than infectious, by spread of the resistance gene to methicillin-resistant *Staphylococcus aureus* strains. In recent years, risk factors for acquiring GRE have been investigated in numerous studies [7–19], but only few have involved situations with strains having genomically identical profiles [7, 13, 15]. We have thus become interested in one GRE outbreak occurring at the *Hôpitaux Universitaires de Strasbourg* (HUS), France. Our study's primary objective was to determine the factors associated with GRE acquisition by cross-transmission during a hospital outbreak. The secondary goal was to describe a hospital GRE outbreak.

## Methods

### Management of GRE

The study involved an outbreak of GRE *faecium* VanB (with genomically identical profiles, verified by the National Reference Centre (CNR) on resistance to antibiotics) that occurred from September 2013 to January 2014 in several wards of HUS, a university hospital with a capacity of approximately 2700 beds.

The outbreak was managed by the Infection Control Team (ICT), along with the help of healthcare workers (HCW). To control this outbreak, we applied rules reflecting current recommendations [20]: Standard precautions associated with the contact precautions (CP) for carrier patients (cases) and for patients having been managed by same nursing team as a case (contact patients). In 2013, at the HUS, the CP for cases involved managing the patient in a private room, dedicated equipment and regular use by the HCW and visitors of disposable gloves, as well as gowns. For contact patients, CP consisted of using dedicated equipment and a disposable apron, but there was no requirement for a private room, gloves or gown. As soon as possible, case patients were transferred to a dedicated unit: for hospitalisation, the dedicated department was a separate unit with only GRE-carriers and dedicated HCW; for chronic haemodialysis, it was a sector with individual rooms but HCW could be shared with non-GRE-carriers. As advised by recommendations: case and contact patients were registered on the information system to be identified as carriers or contacts by the hospital's admissions software; contact patients were screened and considered to be non-GRE carriers after three weekly GRE-negative screening (rectal and/or colostomy swab) following the end of exposure to a case; environmental sampling was recommended only in case of a not ending outbreak.

To highlight the factors associated with acquiring this GRE epidemic strain, we implemented a monocentric, case–control study with individual matching of four controls for one case. The choice to match four controls for one case was based on the possibilities to recruit controls, and on the information from literature showing that the gain in power is negligible beyond four controls for one case [21].

### Definition of cases and controls

The cases were patients at the HUS with at least one positive sample containing the involved strain of GRE *faecium* VanB during this outbreak (infection or colonisation). Each case created contact patients. According to current French recommendations [20], these contact patients are defined as patients having been managed by same nursing team as a case during inpatient hospitalisation (possibly in several wards) or/and haemodialysis sessions. The search period for contact patients was defined based on the last negative screening of the case (or, otherwise, his admission to the HUS) until his management in the dedicated department or until the end of his management (death or discharge from the HUS). Among contact patients of each case, we drew randomly four controls. The controls were contact patients who never had any positive sample with the strain involved and who had at least three negative screenings after exposure to a case, within a minimum time period of 14 days after exposure (weekly screening, according to the recommendations [20]) and not exceeding 6 weeks. Contact patients without GRE screening or with screenings that were excessively late (more than 6 weeks) or incomplete (<three screenings) were excluded from participating to the study.

This study received an approval opinion from the Ethics Committee of the HUS and an approval from the CNIL (Commission Nationale de l'Informatique et des Libertés (National Commission for Information Technology and Civil Liberties)).

### Study parameters

Parameters were selected based on both our experience and the literature (Medline database). A collection grid was completed for each patient (cases and controls), comprising the following data: demographic and clinical; use of antibiotics or antacids over the 3 previous months and during hospitalisation (from admission to screenings); length of hospitalisation and the number of dialysis sessions during the year and in the month preceding the screenings; exposure to a case (presence of diarrhoea, and isolation measures implemented for the case); cross-transmission risk (acute or chronic dialysis, diarrhoea, dependence defined by the need for assistance when washing one's body, management of excretions defined by the use of anatomical protection, the use of a bedpan, existence of stool collection through an ostomy or the ability to go to the bathroom, physical therapy, isolation measures, i.e. protective isolation or CP implemented throughout the duration of exposure).

### Microbiology

GRE screening was performed by swabbing (Copan ESwab) the carrier sites (rectum and/or ostomy), preferably in the morning, before toilette. The sample was cultured in a specific medium (bioMérieux chromID VRETM agar containing 8 mg/l of vancomycin) for 48 h. If suspicious colonies were isolated, an initial search for *vanA* and *vanB* resistance genes was performed using molecular biology directly on the sample (real-time PCR, ABI Prism 7500, Applied Biosystems, Foster City, CA, USA). If this search was negative, no additional tests were carried out. If the result was positive, additional tests were done, namely an identification (mass spectrometer – MALDI-TOF, Bruker) to differentiate the *Enterococcu faecium* and *E. faecalis* strains, along with an antibiogram (Vitek 2, bioMérieux) to assess sensitivity to vancomycin and teicoplanin. PCR was used to confirm the isolated strains. The bacteriology laboratory forwarded the results in the next 2–4 days. The laboratory reported positive results directly to the ICT, and a message was also attached to the result inviting the HCW to contact the ICT. Genomic comparison of the strains was performed by the CNR. This comparison was conducted using the DIVERSILAB^®^ rep-PCR method (bioMérieux).

### Statistical analyses

Statistical analyses were performed using a Bayesian approach [22, 23] with the R software, Version 3.2.2 (copyright The R Foundation for Statistical Computing, Vienna, Austria) and with JAGS software, Version 3.4.0. Univariate analysis was carried out using a mixed logistic regression model including a cluster random effect to account for individual matching between the cases and controls. Parameters were estimated using MCMC (Markov chain Monte Carlo). After a warm-up period of 5000 iterations, 100 000 new iterations were produced to establish the parameter's *posterior* distribution. Convergence of the algorithm to a stationary distribution was assessed graphically. Results were presented as odd ratio (OR), with its respective 95% credibility interval (CI), and the probability that the OR would be higher than one was calculated based on the *posterior* distribution. Variables presenting a probability of being associated with the case's status (Prob OR>1, here in after abbreviated ‘*Pr*’) >0.850, indicating an increased risk of being a case, or lower than 0.150, meaning a reduced risk of being a case (or a protective effect), were included in a multivariate model. It should be noted that these probabilities (*Pr*) must not be confused with the *P*-value of classical statistical analyses. The *Pr* value close to either 0 or 1 is suggested of an effect. Bounds for categorisation of *Pr* were defined at 85% (or 15%), 95% (or 5%) and 99% (or 1%), corresponding with moderate, strong and very strong evidence for an association with the status of the patient.

When available, results from previous studies were used to build informative prior distributions on the log (OR) (Table 1 and appendix). The prior was built by using a Gaussian distribution with average ‘*μ*’ and standard deviation ‘*σ*’, noted *N* (*μ*;*σ*). The average corresponded to the log (OR) originating from the literature and the standard deviation was calculated based on the published confidence interval and weighted by a fixed parameter (between 2 and 4) that determines the amount of historical data to be included in the analysis of the current data. If necessary, hierarchical models were used to combine the effects from different publications. Sensitivity analyses with vague priors were also conducted. When no information was available, we used vague priors centred on the value 0 with a wide standard deviation, *N* (0; 2.6), which corresponds to a prior distribution indicating an OR included with 95% probability in the interval (1/152; 152).

**Table 1.** Informative priors

Reference	Country	Context	Number of cases and controls	Parameters: OR (95% CI)
Servais A, 2009 [13]	France	Epidemic* (identical strains)	14/125	Gender (male): 9.0 (1.3–388.0) Diabetes: 1.7 (0.5–5.9) Glycopeptides the previous month: 11.1 (1.3–90.0) Fluoroquinolone the previous month: 5.0 (0.4–38.9) Dialysis: 9.6 (2.0–90.9) Diarrhoea: 2.3 (0.1–25.8)
MacIntyre CR, 2001 [11]	Australia	Epidemic*	19/66	Vancomycin during hospitalisation: 1.1 (0.4–3.4) Metronidazole during hospitalisation: 4.4 (1.5–13.4) Dialysis: 1.0 (0.2–5.1)
McEvoy SP, 2006 [10]	Australia	Epidemic*	107/107	Gender (male): 0.86 (0.50–1.47) Diabetes: 3.98 (1.97–8.04) Chronic renal insufficiency: 4.82 (2.00–11.64) C3G during hospitalisation: 3.43 (1.83–6.44) Vancomycin during hospitalisation: 2.88 (0.99–8.40) Metronidazole during hospitalisation: 2.93 (1.43–6.01) Fluoroquinolone during hospitalisation: 2.36 (0.86–6.45) Age: 1.03 (1.01–1.05)
Karki S, 2012 [15]	Australia	Prevalence*	58/116	Gender (male): 0.92 (0.46–1.88) Antibiotics during hospitalisation: 3.83 (1.79–8.54) Metronidazole during hospitalisation 0.48 (0.13–1.43) Diarrhoea: 2.54 (0.94–6.85)
Hoshuyama T, 2008 [7]	Japan	Epidemic* (identical strains)	14/45	Chemotherapy: 1.7 (0.4–5.7) Prolonged bed rest: 4.1 (1.07–15.5)

*Enterococcus faecium vanB epidemic.

## Results

### The outbreak

**Fig. 1.**
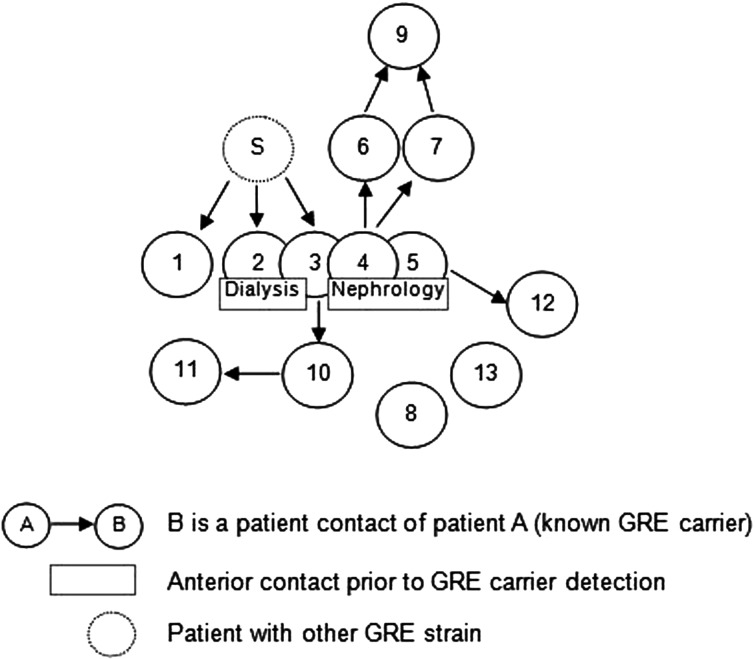
Links between cases. Direct or indirect links between the patients before or during their glycopeptide-resistant enterococci (GRE) carriage. The case ‘S’ had a link with the cases #1, #2 and #3 but it was carrier of another GRE strain according to the genetic comparison. For the cases #8 and #13, no link was found.

**Fig. 2.**
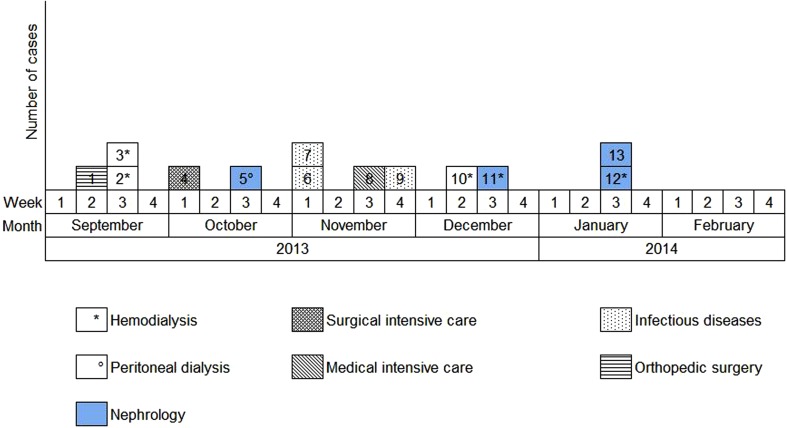
Epidemic curve. New cases of glycopeptide-resistant enterococci (single-strain) per week from September 2013 to February 2014 and wards in which the cases were discovered (wards that took sample).

Overall, we were able to identify 13 patients carrying genetically identical GRE *faecium* VanB between September 2013 and January 2014. These 13 cases were distributed among several hospital wards, mostly nephrology (54%) and infectious diseases (23%) (Table 2). Seven cases were identified fortuitously (Table 3). Indeed they were not screened in the follow-up of contact patients of this outbreak (because they were not identified as a contact, i.e. as a patient managed by same nursing team as a case of this outbreak). But four of these cases (cases #2, #3, #4, #5) had previously been hospitalised together before their screening was found positive, precisely in dialysis and nephrology wards. In total, 10 cases have been transferred in the dedicated department after their identification. Figures 1 and 2 show chronology of events and links between cases.

**Table 2.** Distribution of carriers during GRE *faecium* VanB outbreak according to the hospital department

Hospital department	Number of cases (%)
Nephrology	7 (53.8)
*On chronic dialysis*	*3 (23.1)*
Infectious diseases	3 (23.1)
Surgical intensive care	1 (7.7)
Medical intensive care	1 (7.7)
Orthopaedic surgery	1 (7.7)
TOTAL	13

**Table 3.** Detection circumstances of carriers during GRE *faecium* VanB outbreak and sampling types

Circumstances of case detection	Sampling type	Number of cases (%)	Identification of cases
Follow-up of contact patients	Screening of digestive GRE carriers	6 (46.2)	#6, #7, #9, #10, #11, #12
Fortuitous discovery	Screening of digestive carriers of another GRE strain	4 (30.8)	#1, #2, #3, #13
	Systematic screening at intensive care unit	2 (15.4)	#4, #8
	Clinical sampling	1 (7.7)	#5
TOTAL		13	

### Population characteristics and statistical analyses

Sixty-five patients were included in the study (13 cases and 52 controls). The two principal reasons for hospitalisation were kidney disease (19/65, or 29%) and infection (15/65, or 23%). Most patients came from their homes (91%). Table 4 shows the population characteristics and results from univariate analyses. In univariate analyses, many parameters have indicated a probability of being associated with the case's status >0.850 (including or without informative priors): diabetes, chronic renal insufficiency, antibiotherapy during the last 3 months, antacids intake during hospitalisation, antibiotherapy during hospitalisation, acute or chronic dialysis session, management of excretions, age and number of hospitalisation days in the year (particularly in nephrology). Several factors have shown a probability of being associated with the case's status lower than 0.150: corticosteroid therapy, immunosuppressive treatment, metronidazole intake during the last 3 months and isolation measures.

**Table 4.** Results of univariate analysis

Characteristics	Cases (*n* = 13)		Controls (*n* = 52)		OR	(95% CI)	Prob OR>1*
**Qualitative variables (*n* (%))**							
Gender (male)	8	(61.5)	29	(55.8)	0.72	(0.20–2.43)	0.308
		*With informative priors*			0.91	(0.34–2.38)	0.421
***Antecedents - setting***							
Ongoing cancer treatment	1	(7.7)	2	(3.8)	1.64	(0.10–17.42)	0.650
		*With informative priors*			1.85	(0.26–11.63)	0.738
Neutropenia (neutrophiles <2G/L)	0	(0.0)	1	(1.92)	0.35	(0.00–12.65)	0.299
Corticosteroid therapy	1	(7.7)	14	(26.9)	0.20	(0.02–1.15)	0.038
Immunosuppressive treatment	1	(7.7)	12	(23.1)	0.25	(0.02–1.40)	0.063
HIV	0	(0.0)	2	(3.8)	0.24	(0.00–5.32)	0.203
Chronic alcoholism or drug addiction	1	(7.7)	8	(15.4)	0.41	(0.03–2.47)	0.180
Diabetes (types 1 or 2)	7	(53.8)	19	(36.5)	1.86	(0.54–6.27)	0.844
		*With informative priors*			2.32	(0.65–6.48)	0.950
Chronic renal insufficiency	9	(69.2)	25	(48.1)	2.25	(0.64–8.43)	0.896
		*With informative priors*			3.45	(1.17–11.05)	0.986
Antibiotherapy during the last 3 months	8	(61.5)	15/51	(29.4)	3.44	(1.02–12.32)	0.976
Third-generation cephalosporines	4	(30.8)	4/51	(7.8)	4.81	(0.97–23.72)	0.972
Carbapenems	2	(15.4)	1/51	(2.0)	6.87	(0.66–83.53)	0.946
Glycopeptides	5	(38.5)	1/51	(2.0)	24.16	(3.74–242.48)	>0.999
		*With informative priors*			27.59	(5.13–196.74)	*>0*.*999*
Fluoroquinolones	4	(30.8)	7/51	(13.7)	2.59	(0.59–10.79)	0.893
		*With informative priors*			3.09	(0.71–13.01)	0.934
Metronidazole	0	(0.0)	3/51	(5.9)	0.17	(0.00–3.21)	0.138
Others	5	(38.5)	10/51	(19.6)	2.37	(0.60–8.79)	0.897
***During hospitalisation***							
Carrier of another multidrug resistant bacterium	5	(38.5)	17	(32.7)	1.18	(0.32–4.14)	0.602
Antacids intake	12	(92.3)	36	(69.2)	3.12	(0.76–17.15)	0.939
Antibiotherapy	13	(100.0)	37	(71.2)	5.63	(1.10–44.02)	0.982
		*With informative priors*			5.85	(1.71–21.57)	0.998
Third-generation cephalosporines	7	(53.9)	16	(30.8)	2.39	(0.70–8.14)	0.919
		*With informative priors*			3.06	(1.25–7.64)	0.992
Carbapenems	4	(30.8)	9	(17.3)	1.94	(0.45–7.71)	0.817
Glycopeptides	8	(61.5)	20	(38.5)	2.34	(0.70–8.16)	0.916
		*With informative priors*			2.41	(0.84–7.22)	0.951
Fluoroquinolones	6	(46.2)	18	(34.6)	1.50	(0.42–5.19)	0.741
		*With informative priors*			1.81	(0.61–5.32)	0.862
Metronidazole	1	(7.7)	5	(9.6)	0.67	(0.05–4.47)	0.351
		*With informative priors*			1.08	(0.19–5.00)	0.537
Others	10	(76.9)	25	(48.1)	3.18	(0.88–13.28)	0.961
***Exposure***							
Dialysis (acute or chronic)	8	(61.5)	22	(42.3)	2.07	(0.59–7.48)	0.872
		*With informative priors*			2.40	(0.68–9.40)	0.914
Diarrhoea	3	(23.1)	14	(26.9)	0.74	(0.15–2.80)	0.334
		*With informative priors*			1.29	(0.42–3.64)	0.674
Dependence	8	(61.5)	27	(51.9)	1.36	(0.40–4.91)	0.688
		*With informative priors*			2.61	(0.80–8.89)	0.944
Pelvis/ostomy/protection	8	(61.5)	22	(42.3)	2.00	(0.58–6.88)	0.864
Physiotherapy sessions	4	(30.8)	9	(17.3)	1.96	(0.45–7.60)	0.825
Isolation measures	0	(0.0)	13	(25.0)	0.06	(0.00–0.59)	0.006
Diarrhoea present in cases	4/8	(50.0)	22	(42.3)	1.16	(0.25–5.31)	0.579
Isolation measures for cases	1/8	(12.5)	17	(32.7)	0.29	(0.02–1.83)	0.100
**Quantitative variables: median (first and third quartiles)**							
Age	71	(63–79)	67	(59–76)	1.01	(1.00–1.04)	0.669
		*With informative priors*			1.03	(0.99–1.08)	0.898
Number of hospitalisation days in the year	68	(57–149)	49	(26–70)	1.02	(1.01–1.03)	0.999
Nephrology	0	(0–49)	0	(0–23)	1.02	(1.00–1.04)	0.980
Intensive care unit	0	(0–10)	0	(0–9)	1.00	(0.97–1.03)	0.569
Number of hospitalisation days in the month	28	(16–31)	27	(7–31)	1.01	(0.96–1.07)	0.704
Nephrology	2	(0–9)	0	(0–4)	1.02	(0.96–1.08)	0.725
Intensive care unit	0	(0–3)	0	(0–4)	1.00	(0.92–1.07)	0.486
Number of haemodialysis sessions in the year	0	(0–9)	0	(0–9)	1.00	(0.98–1.01)**	0.353
Number of haemodialysis sessions in the month	0	(0–5)	0	(0–2)	1.00	(0.90–1.10)**	0.509

*Probability that the OR would be higher than one.

**Risk ratio (RR).

Following multivariate analysis including informative priors when the latter were available (Table 5), the intake of antibiotics during hospitalisation (OR = 3.52 (0.94–13.62) with a probability *Pr* that this parameter is associated with the case's status equal to 0.968), the number of hospitalisation days during the previous year (OR = 1.15 (0.99–1.37), *Pr* = 0.964) and antacids intake (OR = 2.87 (0.50–19.78), *Pr* = 0.878) were associated with a higher risk of carrying epidemic GRE. Two protective factors were revealed by this analysis: isolation of the patient during exposure to a case (OR = 0.03 (0.00–0.40), *Pr* = 0.003) and immunosuppression defined by the intake of corticosteroids and/or immunosuppressive treatment (OR = 0.10 (0.01–1.01), *Pr* = 0.026).

**Table 5.** Results of multivariate analysis with informative priors

Characteristics	OR	(95% CI)	Prob OR>1*
Corticosteroid therapy + immunosuppressive treatment	0.10	(0.01–1.01)	0.026
Diabetes (types 1 and 2)	1.71	(0.53–5.60)	0.812
Age	0.98	(0.95–1.02)	0.182
Antibiotherapy during the last 3 months	1.87	(0.38–9.34)	0.784
Number of hospitalisation days in the year	1.15	(0.99–1.37)	0.964
Antacids intake during hospitalisation	2.87	(0.50–19.78)	0.878
Antibiotherapy during hospitalisation	3.52	(0.94–13.62)	0.968
Pelvis/ostomy/protection	1.15	(0.30–7.54)	0.691
Isolation measures	0.03	(0.00–0.40)	0.003

*Probability that the OR would be higher than one.

## Discussion

Besides the parameters classically found in the literature, we sought to further determine the favouring or protective factors associated with acquiring GRE during an outbreak. Following multivariate analysis, and after considering informative priors, the factors shown to impact were the number of hospitalisation days in the previous year, antacids intake and intake of antibiotics during hospitalisation. Immunosuppression and isolation measures have shown a protective effect.

Some of these parameters were already known impact factors, such as prior hospitalisation during the year [12–14, 16] and antibiotic therapy during hospitalisation [7, 15, 24], but these parameters were not always defined the same way. In fact, sometimes hospitalisation history was a qualitative variable, sometimes a quantitative variable (number of hospitalisation days). We have preferred quantitative variable to define hospitalisation history as a cross-transmission risk factor because the longer the hospitalisation time, the higher risk of being exposed to a GRE-carrier patient. Regarding antibiotic therapy, they are more or less detailed according to the studies. A recent publication with a different study design highlights the influence of antibiotic consumption quantified as patient antimicrobial days on the horizontal transmission events [24]. Antacids intake showed also a link to carrying GRE. A study has revealed the same result in 2002 [25]. This parameter is rarely searched for while it can affect gastrointestinal function. In fact, by decreasing gastric acidity, antacids may create a medium suitable for colonisation by GRE.

Only very few studies have considered specific cross-transmission parameters. We found a US study conducted in 2003 that highlighted a link between acquiring GRE and placing patients in rooms at risk (GRE-positive environmental samples) [9]. Another US study involving eight hospital wards, carried out in 2001, revealed a link between acquiring GRE and proximity to a GRE-carrier patient without CP (proximity index calculation) [26]. One of the studies utilised for our informative priors demonstrated that being in the same ward with a carrier was the most significant GRE-carrying risk factor [11]. In our study, we were unable to consider this type of information because we did not perform regular environmental sampling and we did not find any link among all cases, whereas they were definitely carriers of the same single GRE strain. Therefore, we were unable to identify one or some of the vectors: environment, HCW, medical device or patient not known as a carrier because he did not undergo screening, or was screened but with false-negative results. In theory, investigations conducted at the time of an alert avoid this, but unfortunately the collected information and conducted screenings are never completely exhaustive, with patients often discharged prior to the triple screening's end.

Moreover, we succeeded in collecting other specific cross-transmission parameters. Some were found in the literature, such as the use of dialysis sessions, dependence, presence of diarrhoea and management of excretions [7, 11, 13, 15]. Others were less specific, such as physical therapy and presence of diarrhoea in index cases. These different parameters did not show any impact on univariate or multivariate analyses, although they were instrumental in increasing the frequency of contacts among patients and personnel, as well as environmental contamination. Finally, it would have been interesting to collect information on the sharing of rooms, nursing staff or medical equipment, though this information proved difficult to collect *a posteriori*. It would thus be more appropriate to collect specific cross-transmission parameters in a prospective manner.

Our study was able to identify protective factors, namely, implementation of isolation measures and immunosuppression (intake of corticosteroids or immunosuppressive treatment). Isolation measures included CP and protective isolation, which appears quite consistent, given that these measures are said to be barriers against the transmission of microorganisms. The significant protective effect of CP was also found by Fossi Djembi *et al*. [17] but they have not made a genomic comparison of their strains. This observation should still increase our confidence and compliance regarding CP for controlling GRE outbreaks. Immunosuppression as a protective factor is a surprising result. Perhaps there could have been a sampling bias associated with the ward where controls came from. However, the controls were drawn randomly among contact patients of each case during the hospital path. Thus, these controls did not come from only ward. Consequently an alternative hypothesis, pertaining to isolation measures (protective isolation or contact protection), must be evoked. It is worth mentioning that among the 15 immunosuppressed controls, only six of them had isolation measures. The comparison with the only immunosuppressed case (which did not have isolation measure) is thus not very relevant. Nevertheless, isolation measures do not seem to be an explanation of the protective effect. Lastly, to evaluate the stability of our results due to the small number of immunosuppressed patients, we ran a sensitivity analysis by using different alternative prior distributions for the OR. Surprisingly, the use of either an optimistic informative prior, a pessimistic informative prior or a lowly informative prior had only little impact on the result, while the addition of a single event among the cases (2/13 instead of 1/13), as part of this sensitivity analysis, led to a disappearance of the effect, suggesting maybe that we lack data to ascertain this result.

Certain parameters showed an effect in the univariate analysis, according to our model's construction algorithm (Pr⩾0.850 or Pr⩽0.150), but were not included in the multivariate analysis. This is the case for parameters linked to nephrology, already documented in the literature and in the description of this outbreak as risk factors for carrying GRE. We made this choice in order to limit the number of parameters in our multivariate analysis, considering our sample size.

It is possible that our study lacks evidence, as it did not allow us to reveal an association between our variable of interest and all of the study's parameters that were selected due to their probable role in GRE transmission. We used a Bayesian approach in order to limit this bias. In fact, Bayesian analysis enables the use of prior information for the parameter of interest, built for instance on previous publications results. In the absence of information, we used a Gaussian vague prior with parameter centred on the value 0 with a wide standard deviation, *N* (0; 2.6). We used data from the literature to be included in the models. These data should be comparable and collected from the literature in a similar way as the data from the study in question. It was thus not always possible to find informative priors for each parameter.

Finally, the description of this outbreak highlighted the importance to detect quickly patients at risk of GRE carriage to implement CP as soon as possible. Indeed none of our cases were in isolation measures before their discovery and many cross-transmissions may have occurred before the knowledge of the carriage. During this period, the patients may have visited different wards, particularly Nephrology wards. After the GRE detection, the rapid transfer to a dedicated department (separated unit with dedicated HCW) no longer has allowed the cross-transmission what has led to the extinction of the outbreak. In France, patients defined at risk of GRE carriage are contact patients and those hospitalised abroad in the previous year. Upon admission, CP are implemented and patients are screened. Others patients do not have preventive measure with regard to the GRE [20].

In our study, we have shown that a history of hospitalisation and the intake of antibiotics and antacids during hospitalisation favour the acquisition of a hospital epidemic strain of GRE while isolation measures are logically protective. The Bayesian approach was particularly useful considering our limited sample size and given that some informative data were available in the literature. Early detection of GRE carriers in order to implement isolation measures and to transfer to dedicated department is an important factor for the management of an outbreak. Systematic search of factors associated with acquisition of GRE could lead to faster implementation of these measures.

## Supplementary material

For supplementary material accompanying this paper visit https://doi.org/10.1017/S0950268818003655. click here to view supplementary material

## References

[1] Leclercq R and Coignard B (2006) Les entérocoques résistants aux glycopeptides: situation en France en 2005. Bulletin Epidémiologique Hebdomadaire 13, 85–87.

[2] Henard S (2011) Control of a regional outbreak of vanA glycopeptide-resistant *Enterococcus faecium*, Eastern France, 2004–2009. International Journal of Hygiene and Environmental Health 214, 265–270. 21330205 10.1016/j.ijheh.2011.01.004

[3] Bourdon N, Fines M and Leclercq R (2008) Caractéristiques des souches d'entérocoques résistants aux glycopeptides isolées en France, 2006–2008. Bulletin Epidémiologique Hebdomadaire 41–42, 391–394.

[4] Stockholm: ECDC (2015) European Centre for Disease Prevention and Control. Antimicrobial resistance surveillance in Europe 2014. *Annual Report of the European Antimicrobial Resistance Surveillance Network* (EARS-Net).

[5] Hautemanière A (2009) A prospective study of the impact of colonization following hospital admission by glycopeptide-resistant enterococci on mortality during a hospital outbreak. American Journal of Infection Control 37, 746–752. 19556034 10.1016/j.ajic.2009.02.007

[6] Salgado CD and Farr BM (2003) Outcomes associated with vancomycin-resistant enterococci: a meta-analysis. Infection Control and Hospital Epidemiology 24, 690–698. 14510253 10.1086/502271

[7] Hoshuyama T (2008) Vancomycin-resistant enterococci (VRE) outbreak at a university hospital in Kitakyushu, Japan: case-control studies. Journal of Infection and Chemotherapy 14, 354–360. 18936888 10.1007/s10156-008-0628-x

[8] Correa AAF (2015) Small hospitals matter: insights from the emergence and spread of vancomycin-resistant enterococci in 2 public hospitals in inner Brazil. Diagnostic Microbiology and Infectious Disease 82, 227–233. 25956931 10.1016/j.diagmicrobio.2015.03.026

[9] Martínez JA (2003) Role of environmental contamination as a risk factor for acquisition of vancomycin-resistant enterococci in patients treated in a medical intensive care unit. Archives of Internal Medicine 163, 1905–1912. 12963563 10.1001/archinte.163.16.1905

[10] McEvoy SP (2006) Risk factors for the acquisition of vancomycin-resistant enterococci during a single-strain outbreak at a major Australian teaching hospital. Journal of Hospital Infection 62, 256–258. 16257091 10.1016/j.jhin.2005.06.018

[11] MacIntyre CR (2001) Risk factors for colonization with vancomycin-resistant enterococci in a Melbourne hospital. Infection Control and Hospital Epidemiology 22, 624–629. 11776348 10.1086/501833

[12] Sakka V (2008) Risk-factors and predictors of mortality in patients colonised with vancomycin-resistant enterococci. Clinical Microbiology and Infection 14, 14–21. 18005178 10.1111/j.1469-0691.2007.01840.x

[13] Servais A (2009) Rapid curbing of a vancomycin-resistant *Enterococcus faecium* outbreak in a nephrology department. Clinical Journal of the American Society of Nephrology 4, 1559–1564. 19713290 10.2215/CJN.03310509 PMC2758257

[14] Assadian O (2007) Prevalence of vancomycin-resistant enterococci colonization and its risk factors in chronic hemodialysis patients in Shiraz, Iran. BMC Infectious Diseases 7, 52. 17553129 10.1186/1471-2334-7-52 PMC1894971

[15] Karki S (2012) Prevalence and risk factors for VRE colonisation in a tertiary hospital in Melbourne, Australia: a cross sectional study. Antimicrobial Resistance and Infection Control 1, 31. 23039285 10.1186/2047-2994-1-31 PMC3523023

[16] Roghmann M (1998) Colonization with vancomycin-resistant enterococci in chronic hemodialysis patients. American Journal of Kidney Diseases 32, 254–257. 9708609 10.1053/ajkd.1998.v32.pm9708609

[17] Fossi Djembi L (2017) Factors associated with vancomycin-resistant Enterococcus acquisition during a large outbreak. Journal of Infection and Public Health 10, 185–190. 27140696 10.1016/j.jiph.2016.04.010

[18] Amberpet R (2016) Screening for intestinal colonization with vancomycin resistant enterococci and associated risk factors among patients admitted to an adult intensive care unit of a large teaching hospital. Journal of Clinical and Diagnostic Research 10, DC06–DC09. 10.7860/JCDR/2016/20562.8418 PMC5071930 27790430

[19] Monteserin N and Larson E (2016) Temporal trends and risk factors for healthcare-associated vancomycin-resistant enterococci in adults. Journal of Hospital Infection 94, 236–241. 27645212 10.1016/j.jhin.2016.07.023 PMC5192559

[20] Instruction DGOS/PF2/DGS/RI1 n°2014-08 du 14 janvier 2014 relative aux recommandations pour la prévention de la transmission croisée des bactéries hautement résistantes aux antibiotiques émergentes.

[21] Ury HK (1975) Efficiency of case-control studies with multiple controls per case: continuous or dichotomous data. Biometrics 31, 643–649. 1100136

[22] Gelman A (2013) Bayesian Data Analysis, 3rd Edn Boca Raton: Chapman and Hall/CRC.

[23] Hoff PD (2009) A First Course in Bayesian Statistical Methods. New York, NY: Springer New York.

[24] Gilbert EM (2017) Factors contributing to vancomycin-resistant Enterococcus spp. horizontal transmission events: exploration of the role of antibacterial consumption. Diagnostic Microbiology and Infectious Disease 89, 72–77. 28669681 10.1016/j.diagmicrobio.2017.05.014

[25] Cetinkaya Y, Falk PS and Mayhall CG (2002) Effect of gastrointestinal bleeding and oral medications on acquisition of vancomycin-resistant *Enterococcus faecium* in hospitalized patients. Clinical Infectious Diseases 35, 935–942. 12355380 10.1086/342580

[26] Byers KE (2001) A hospital epidemic of vancomycin-resistant enterococcus: risk factors and control. Infection Control of Hospital Epidemiology 22, 140–147. 10.1086/501880 11310691

