# Supplementary material for: Factors associated with acquisition of glycopeptide-resistant enterococci during a single-strain outbreak
Source: Epidemiol Infect. 2019 Mar 20;147:e158. doi: 10.1017/S0950268818003655 (PMC6518758; doi:10.1017/S0950268818003655)
Supplement: Supplementary file 1 [file S0950268818003655sup001.docx]

Epidemiology and Infection

**Factors associated with acquisition of glycopeptide-resistant enterococci during a single-strain outbreak**

S. DEBOSCKER*^1,2^, P. SCHNEIDER^3^, F. SEVERAC^2,4^, C. MENARD^5^, J. GAUDART^6,7^, T. LAVIGNE^1,8^, N. MEYER^2,4^

**Supplementary Material**

Informative priors and posterior Beta distribution

| **Parameters** | **Prior and posterior Beta distribution** | **Reference  (informative priors)** |
| --- | --- | --- |
| Gender (male) | 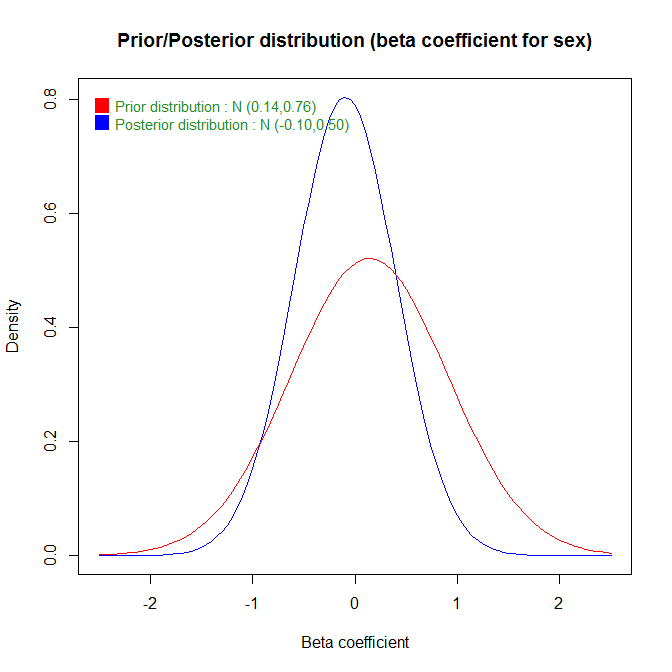 | Servais A, 2009 [13]  McEvoy SP, 2006 [10]  Karki S, 2012 [15] |
| Chemotherapy (Ongoing cancer treatment) | 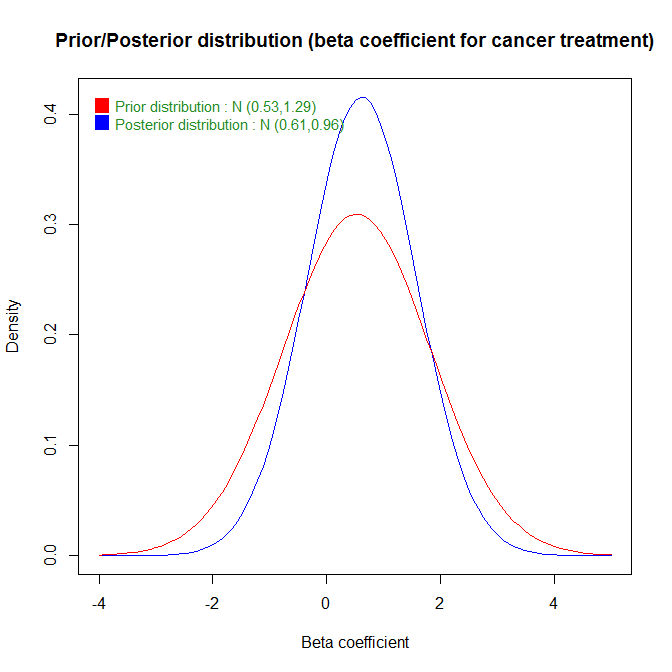 | Hoshuyama T, 2008 [7] |
| Diabetes | 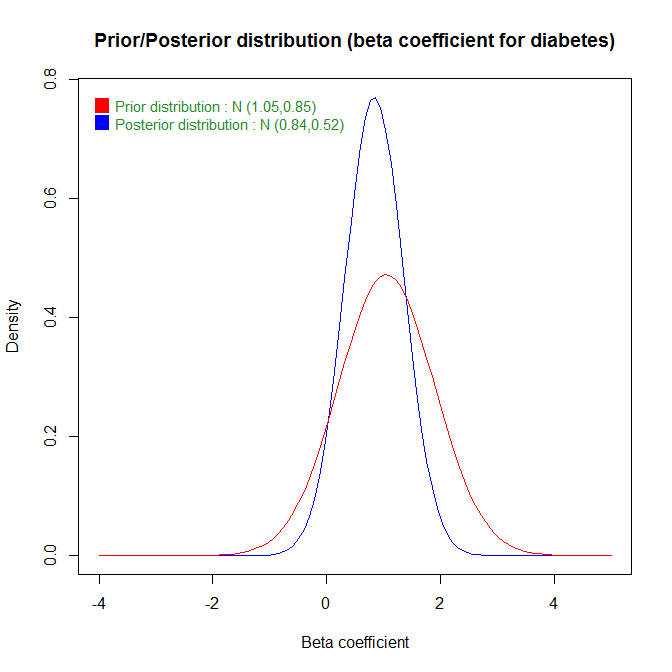 | Servais A, 2009 [13]  McEvoy SP, 2006 [10] |
| Chronic renal insufficiency | 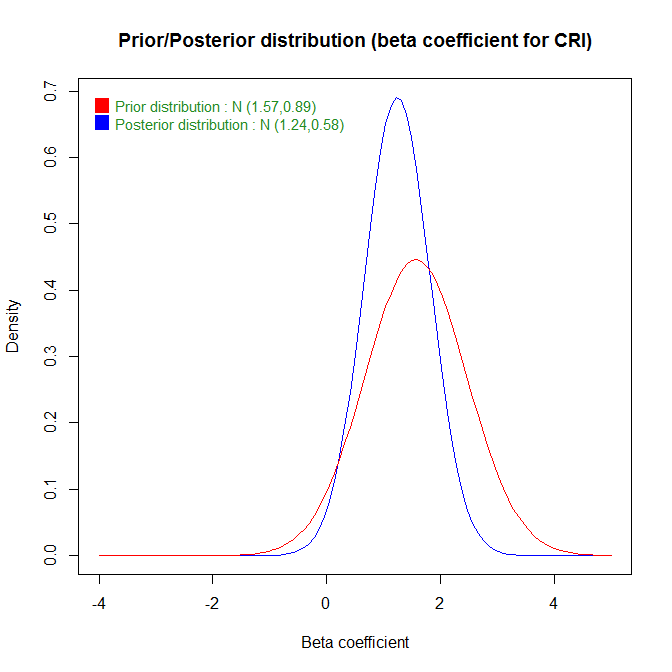 | McEvoy SP, 2006 [10] |
| Glycopeptides the previous month | 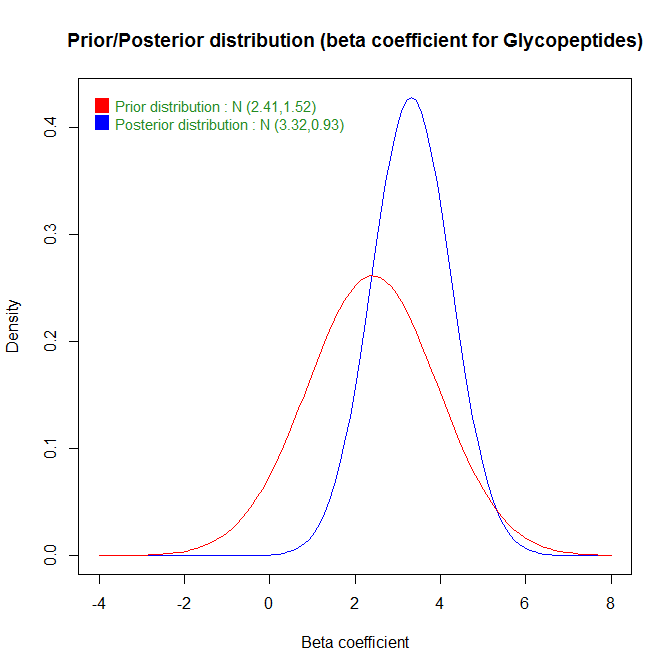 | Servais A, 2009 [13] |
| Fluoroquinolone the previous month | 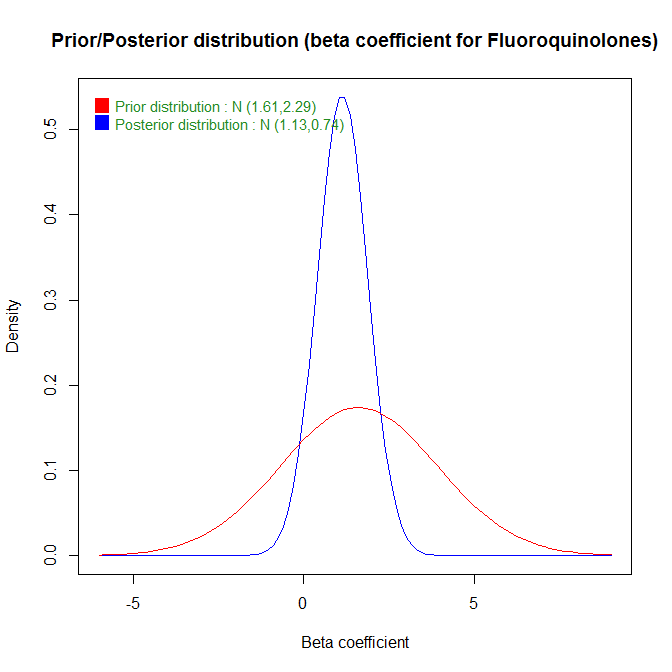 | Servais A, 2009 [13] |
| Antibiotics during hospitalization | 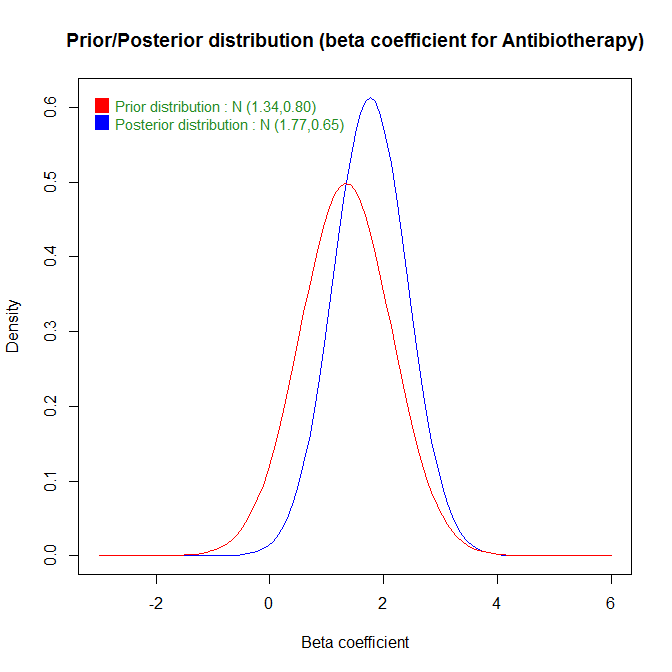 | Karki S, 2012 [15] |
| C3G during hospitalization | 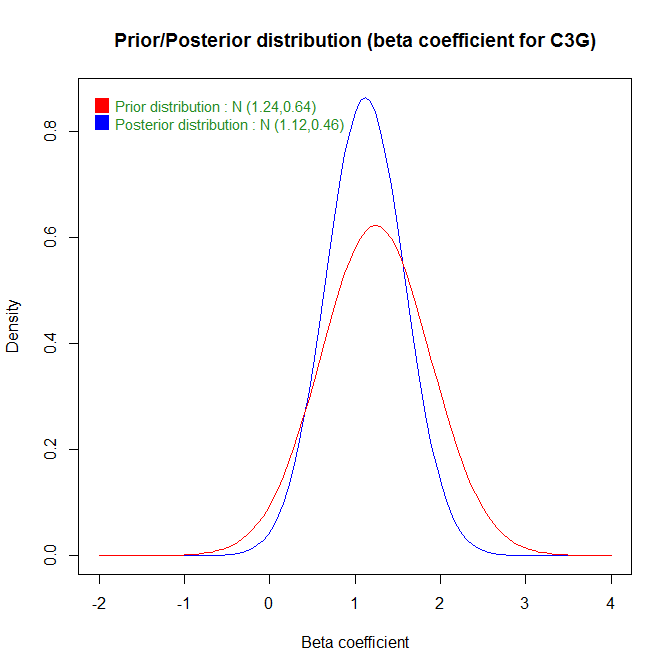 | McEvoy SP, 2006 [10] |
| Vancomycin during hospitalization | 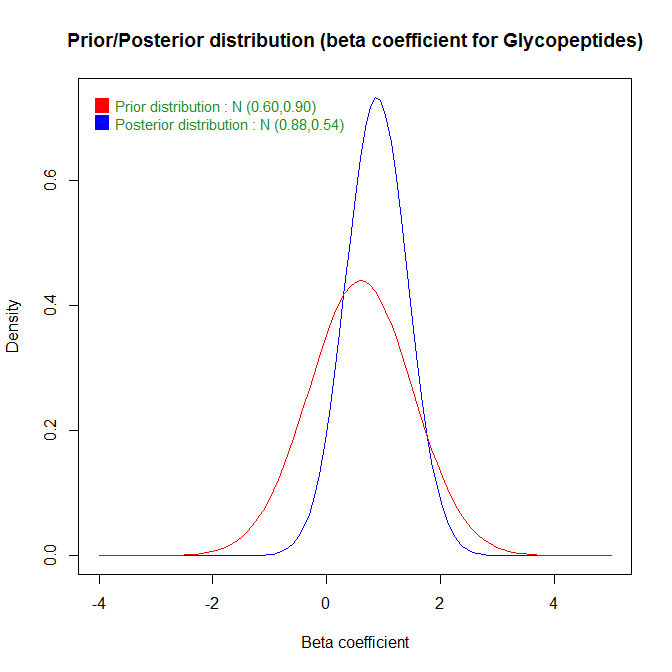 | MacIntyre CR, 2001 [11]  McEvoy SP, 2006 [10] |
| Fluoroquinolone during hospitalization | 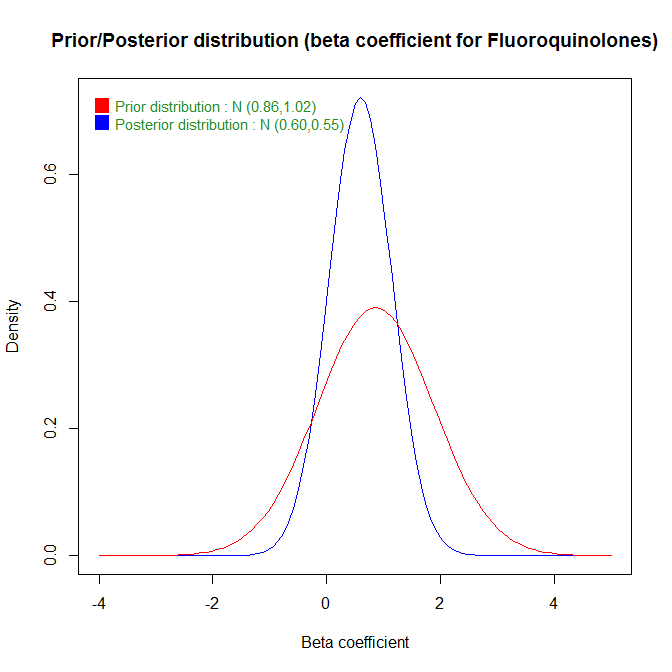 | McEvoy SP, 2006 [10] |
| Metronidazole during hospitalization | 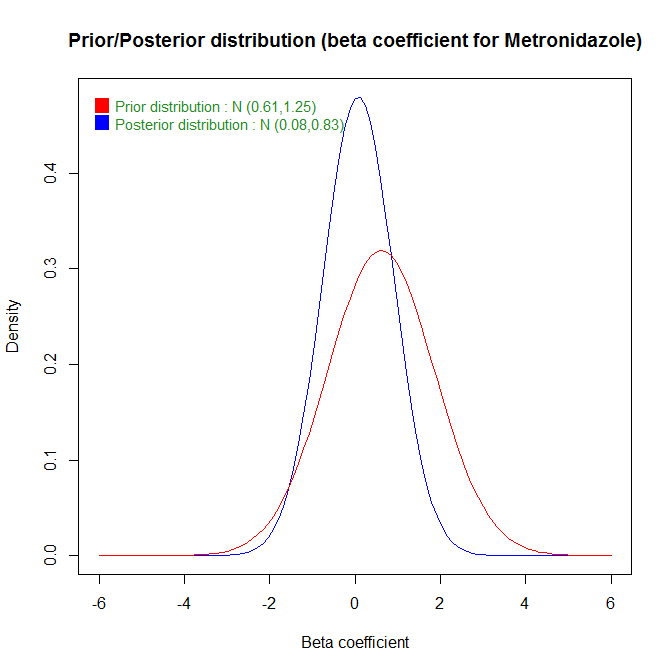 | MacIntyre CR, 2001 [11]  McEvoy SP, 2006 [10]  Karki S, 2012 [15] |
| Dialysis | 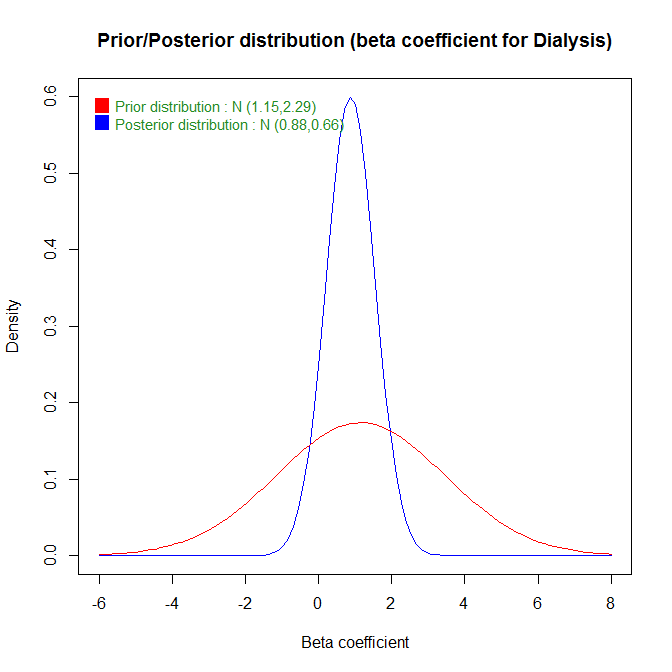 | Servais A, 2009 [13]  MacIntyre CR, 2001 [11] |
| Diarrhoea | 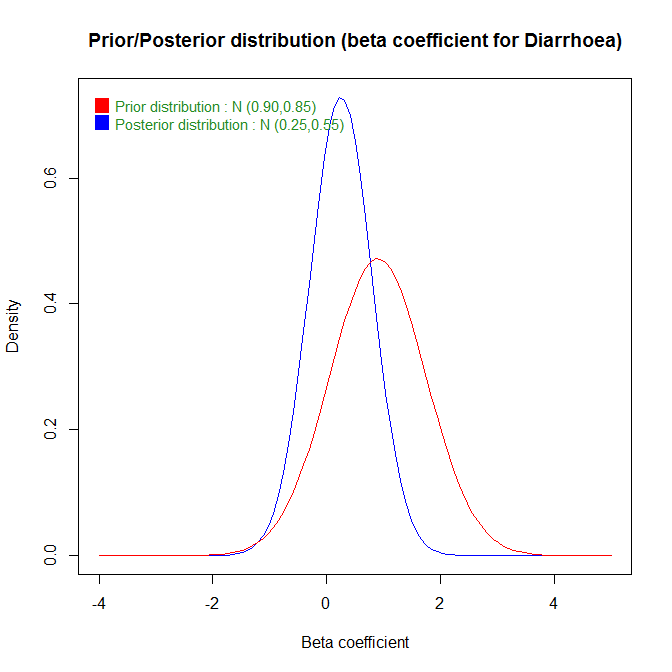 | Servais A, 2009 [13]  Karki S, 2012 [15] |
| Prolonged bed rest (dependence) | 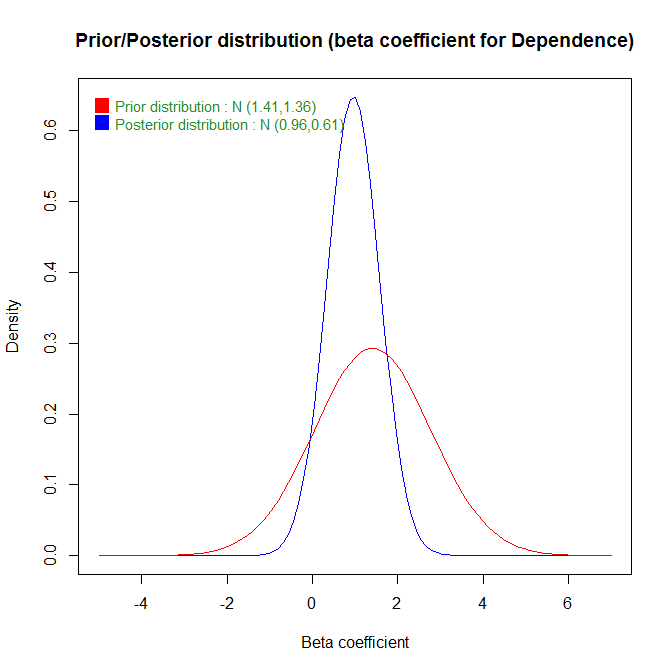 | Hoshuyama T, 2008 [7] |
| Age | 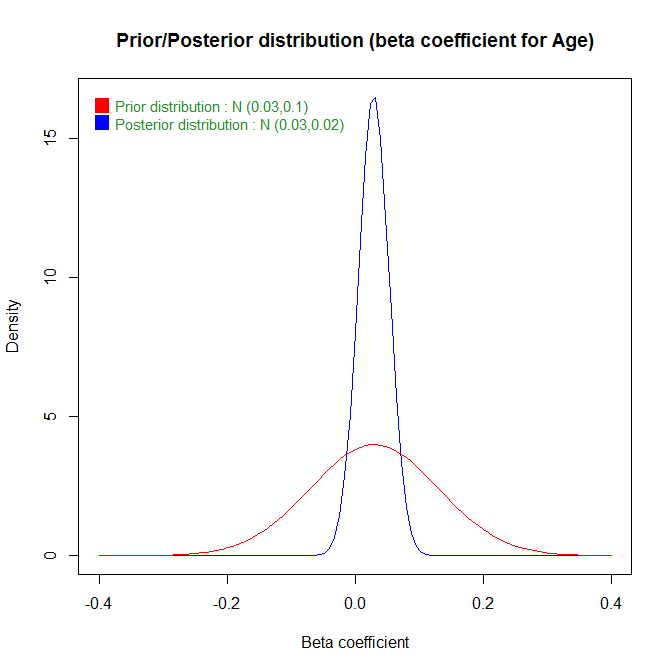 | McEvoy SP, 2006 [10] |
